# Effects of Continuous Positive Airway Pressure on Sleep EEG Characteristics in Patients with Primary Central Sleep Apnea Syndrome

**DOI:** 10.1155/2021/6657724

**Published:** 2021-04-22

**Authors:** Cheng Zhang, Kun Chen, Guangfa Wang, Jue Zhang, Jing Ma

**Affiliations:** ^1^Department of Respiratory and Critical Care Medicine, Peking University First Hospital, Beijing 100034, China; ^2^Academy of Advanced Interdisciplinary Studies, Peking University, Beijing 100871, China

## Abstract

This study aimed to investigate the effects of continuous positive airway pressure (CPAP) on the electroencephalographic (EEG) characteristics of patients with primary central sleep apnea syndrome (CSAS). Nine patients with primary CSAS were enrolled in this study. The raw sleep EEG data were analyzed based on two main factors: fractal dimension (FD) and zero-crossing rate of detrended FD. Additionally, conventional EEG spectral analysis in the delta, theta, alpha, and beta bands was conducted using a fast Fourier transform. The FD in patients with primary CSAS who underwent CPAP treatment was significantly decreased during nonrapid eye movement (NREM) sleep but increased during rapid eye movement (REM) sleep (*p* < 0.05). Regarding the EEG spectral analysis, the alpha power increased, while the delta/alpha ratio decreased during REM sleep in patients with CSAS (*p* < 0.05). In conclusion, CPAP treatment can reduce FD in NREM sleep and increase FD during REM sleep in patients with primary CSAS. FD may be used as a new biomarker of EEG stability and improvement in brain function after CPAP treatment for primary CSAS.

## 1. Introduction

Central sleep apnea syndrome (CSAS) is a respiratory disorder that occurs when the respiratory center fails to issue the respiratory drive effectively due to various causes [1]. CSAS encompasses a wide range of diseases and covers eight categories according to the International Classification of Sleep Disorders-Third Edition (ICSD-3) [2], such as central sleep apnea (CSA) associated with Cheyne–Stokes respiration, CSA due to drugs or substances, and primary CSA. Except for primary CSA, most CSAs are caused by various underlying diseases or other medical conditions, for example, the most common is Cheyne–Stokes respiration caused by heart failure or stroke.

Compared with obstructive sleep apnea (OSA), CSAS has a lower prevalence in the general population [1]. Both OSA and CSA are characterized by recurrent nocturnal hypoxia and arousals, which are associated with daytime sleepiness, inattention, memory loss, and other signs of impaired brain function. Meanwhile, continuous positive airway pressure (CPAP) has been shown to improve brain function in patients with OSA [3]. Electroencephalography (EEG) is a tool for evaluating brain function. Quantitative EEG analysis has become an important method for assessing brain function in various populations [4, 5]. Previous studies have shown that CPAP can improve sleep EEG features in patients with OSA [6–9], such as correcting EEG slowing and reducing the complexity and increasing the stability of sleep EEG waves, which may explain the improvement of brain function in patients with OSA who receive CPAP treatment. However, the efficacy of CPAP in treating patients with CSAS and its effects on sleep EEG are not yet known.

The most common method for quantitative EEG analysis is EEG power spectral analysis [6, 7, 10], which includes the analysis of the absolute and relative power of delta, theta, alpha, and beta frequency bands. Fractal dimension (FD) is a new method for measuring the irregularity and complexity of an object. It was initially introduced as a description of self-similar objects [11] and was subsequently utilized in a variety of scientific disciplines [12, 13]. FD is more suitable for the analysis of nonlinear and nonstationary physiological data, such as EEG [13]. In our previous study [13], we found that FD could reflect the sleep macroarchitecture of each participant. Furthermore, the fast fluctuation of FD, as measured by the zero-crossing rate of detrended FD (zDFD), is a useful indicator of sleep disturbance. CPAP can decrease FD in nonrapid eye movement (NREM) sleep and zDFD in both NREM and rapid eye movement (REM) sleep in patients with OSA [9].

Therefore, the purpose of this study was to explore the effect of CPAP treatment on the sleep EEG of patients with CSAS using FD and conventional EEG spectral analysis. We attempted to identify markers from these EEG signal analyses that could reflect improved brain function in patients with CSAS undergoing CPAP treatment. To avoid the interference of underlying diseases and potential confounding effects, we included only primary CSAS patients.

## 2. Materials and Methods

### 2.1. Study Participants

In this retrospective study, patients aged ≥20 years who were diagnosed with primary CSAS in the sleep lab of Peking University First Hospital and received CPAP pressure titration were enrolled.

According to ICSD-3, the diagnosis criteria of primary CSAS were as follows:

(A) At least one of the following:
  1. Sleepiness
  2. Difficulty initiating or maintaining sleep, frequent awakenings, or nonrestorative sleep
  3. Awakening with shortness of breath
  4. Snoring
  5. Witnessed apneas
(B) PSG demonstrates all of the following:
  1. Five or more central apneas and/or central hypopneas per hour of sleep
  2. The number of central apneas and/or central hypopneas is >50% of the total number of apneas and hypopneas
  3. Absence of Cheyne–Stokes breathing
(C) No evidence of daytime or nocturnal hypoventilation
(D) The disorder is not better explained by another current sleep disorder, medical or neurological disorder, medication use, or substance use disorder

Finally, nine patients with primary CSAS, in accordance with the above criteria, who underwent full-night CPAP titration were included.

This retrospective study was approved by the ethics committee of Peking University First Hospital, and the requirement for obtaining informed consent was waived (Ethics Approval No. 2017 [1363]). The study adhered to the Declaration of Helsinki, and patient confidentiality was maintained.

### 2.2. Study Methods

#### 2.2.1. Overnight PSG

Sleep apnea was confirmed by overnight PSG (Compumedics, E-Series, Australia). Six channels of EEG signals (C3-M2, C4-M1, F3-M2, F4-M1, O1-M2, and O2-M1), two channels of electrooculography signals (E1-M2 and E1-M2), and chin EMG (EMG1–EMG2, EMG1–EMG3), electrocardiography, respiration (nasal pressure, airflow), oxygen saturation, abdominal and chest movement, and leg movements were recorded according to the American Academy of Sleep Medicine (AASM) scoring manual (version 2.4) [14].

Sleep stage and respiratory events were analyzed according to the guidelines of the AASM [14]. Sleep stages were divided into *N*1, *N*2, *N*3, *R*, and *W* stages. Respiratory events were divided into obstructive apnea, central apnea, mixed apnea, and hypopnea. The apnea-hypopnea index (AHI) was defined and calculated as the sum of the number of apneas and hypopneas per hour.

### 2.3. CPAP Titration

CPAP titration was conducted according to the CPAP titration guidelines [15].

### 2.4. Calculation and Analysis of FD and zDFD

The FD and zDFD indices of the EEG before and after CPAP titration were analyzed. Briefly, FD is an engineering index used to describe the complexity of an EEG. Furthermore, the fast fluctuation of FD was measured using zDFD.

All raw FD and zDFD data were analyzed by the Academy for Advanced Interdisciplinary Studies, Peking University, and the single channel of C3-M2 was used for analysis. The FD and zDFD were calculated using custom programming in MATLAB (MathWorks, Inc., Natick, MA).

The general calculation processing is as follows: (1) the local FD of sleep EEG signals in each 30 s epoch was estimated for all patients using a standard “box-counting” algorithm. FD was defined as the following equation.

(1) $$\begin{array}{c} \text{FD}=\;-\lim_{r⟶0}\frac{\log_{2}\left[N\left(r\right)\right]}{\log_{2}\left(r\right)}. \end{array}$$

By covering a structure such as an EEG signal with boxes of side length *r*, the FD is given, where *N*(*r*) is the number of nonempty boxes needed to completely cover the structure, and FD corresponds to the slope of the plot versus log2*N*(*r*). An FD time series was generated by sequentially moving the 30 s window forward in time. We applied an adaptive data analysis technique, called the empirical mode decomposition (EMD) algorithm, to detrend the FD sequence. In this study, the EMD algorithm was employed to smooth the FD sequences of the full-night EEG in an adaptive manner. The detailed processing method has been described in our previous reports [10].

### 2.5. EEG Spectral Analysis

Quantitative EEG analysis was conducted [16, 17]. All EEG study sampling rates were >200 Hz. A standard fast Fourier transform (FFT) with a Hamming window was performed. The FFT was applied to contiguous 30 s segments, and periodograms were calculated from one successive segment to obtain the absolute power in every stage lasting 30 s for the delta (0.1–3.99 Hz), theta (4–7.99 Hz), alpha (8–13 Hz), and beta (13–32 Hz) frequency bands of the central (C3-M2) EEG. Relative power was also calculated.

### 2.6. Statistical Methods

The SPSS software package, version 17.0 (SPSS, Inc., Chicago, IL, USA), was used for statistical analyses. Normality tests were also performed. Data conforming to a normal distribution were described as mean ± standard deviations. The FD, zDFD, and relative power percentages of the delta, theta, alpha, and beta waves before and after CPAP treatment were compared using a *t*-test of two related samples. A *p* value <0.05 was considered statistically significant.

## 3. Results

Nine CSAS patients (male:female ratio of 8 : 1; age, 60.2 ± 10.8 years; body mass index, 28.4 ± 4.0 kg/m^2^) were enrolled. After the initial monitoring, the nine CSAS patients underwent manual CPAP titration within 1 month. CPAP decreased the AHI effectively for patients with CSAS (from 60.0 ± 17.0 times/h to 6.0 ± 7.1 times/h; *p* < 0.05). The demographic and basic data are given in Table 1.

CPAP had a significant effect on sleep architecture in patients with CSAS. After the application of CPAP, the proportion of light sleep (*N*1 + *N*2 sleep) decreased, and slow-wave sleep (*N*3 sleep) and REM sleep increased (Table 2); however, some of the changes showed no statistical differences probably owing to the relatively small sample size.

**Figure 1:**
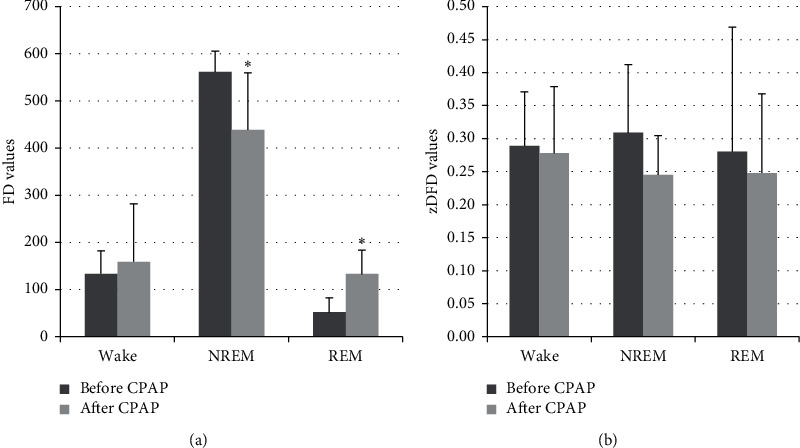
The changes of fractal dimension (FD) and zero-crossing rate of detrended FD (zDFD) in patients with primary central sleep apnea syndrome (CSAS) before and after continuous positive airway pressure (CPAP) treatment. (a) The FD significantly decreased in nonrapid eye movement (NREM) sleep but increased in rapid eye movement (REM) sleep with CPAP treatment (^*∗*^*p* < 0.05; before CPAP). (b) There were no significant changes in zDFD before and after CPAP treatment in either NREM or REM sleep.

After CPAP treatment, the FD decreased significantly during NREM sleep but increased significantly during REM sleep in patients with CSAS (*p* < 0.05; Figure 1). The zDFD in these patients showed a downward trend, although this difference was not statistically significant (Figure 1). The data are presented in Table 3.

**Figure 2:**
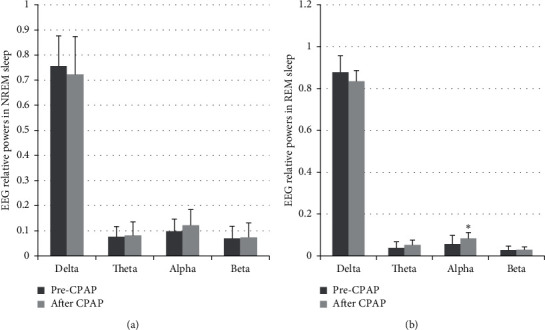
The changes of sleep EEG spectral power before and after CPAP in patients with CSAS in nonrapid eye movement (NREM) sleep (a) and rapid eye movement (REM) sleep. (b) ^*∗*^*p* < 0.05 vs. before CPAP.

Regarding the EEG spectral analysis results, the alpha power increased and delta/alpha ratio decreased during REM sleep in patients with CSAS after CPAP treatment (*p* < 0.05). There were no significant changes in delta, theta, and beta activities in patients with CSAS after CPAP treatment, although delta activity tended to decrease, while theta and alpha activities increased (Figure 2; *p* > 0.05).

## 4. Discussion

This study investigates the efficacy of CPAP on primary CSAS from the EEG perspective. Engineering parameters, FD and zDFD, and conventional EEG spectral analysis were used to analyze sleep EEG before and after CPAP. Based on the results, CPAP treatment could reduce the FD of NREM sleep in patients with primary CSAS, thereby reducing the complexity of EEG and stabilizing the EEG. On the other hand, CPAP could increase the FD of REM sleep, which may increase the EEG activity and improve the brain function. Conventional spectral power analysis showed that with CPAP treatment, the alpha power increased while the delta/alpha ratio decreased during REM sleep, suggesting increased EEG activity, which is consistent with the results of FD.

The impairment of brain function in patients with sleep breathing is an evident and prominent problem. Patients with sleep apnea often complain of daytime sleepiness, memory loss, and attention deficit, and CPAP is an effective treatment method for these patients. Until now, there has been limited research regarding the mechanism of CPAP efficacy on central sleep apnea [1, 15]. Since the central sleep apnea is very complicated and include a variety of diseases, the present study chose patients with primary CSAS to exclude the potential confounding effect.

Researchers have shown that EEG, assessed by quantitative analysis, is a tool to evaluate brain dysfunctions [10]. The most common method used in these quantitative analyses of EEG studies is power spectral analysis [6, 7, 10]. FD is a new parameter more suitable for the analysis of nonlinear and nonstationary physiological data, such as EEG [13]. In our previous study, we showed that the variation in FD reflects the macrostructure of sleep and that the fast fluctuation of FD, as measured by the zDFD, is a useful indicator of sleep disturbance; hence, it correlates with the AHI [13]. Sleep stages were divided into *N*1, *N*2, *N*3, *R*, and *W* stages. NREM sleep stage included *N*1, *N*2 (light sleep), and *N*3 (deep sleep). During deep sleep, the synchronization of EEG activity is significantly enhanced. The REM period is a period of paradoxical sleep, with weak muscle activity and active EEG. In our previous study [13], we showed that the FD value decreased from *W* to *N*1, *N*2, and *N*3 but increased during REM sleep, which showed that FD is a good indicator of the complexity of EEG. The zDFD reflects the fast fluctuation of FD in a certain period, which indicates the variability of EEG complexity. We used the FD and zDFD to analyze the effects of CPAP therapy on sleep EEG in patients with OSAS [9]. After CPAP treatment, the FD of EEG decreased significantly in NREM sleep, while the FD of EEG increased significantly in REM sleep.

Similar to the effect of CPAP on OSA patients, the present study showed that FD of EEG decreased significantly during NREM sleep in CSAS patients with CPAP. As mentioned earlier, the FD can reflect the complexity of the EEG. Thus, the results suggest that CPAP therapy can reduce the complexity of sleep EEG in patients with CSAS to achieve a more stable EEG pattern. On the other hand, the FD of sleep EEG increased during REM sleep in patients with CSAS. The EEG slowing in REM sleep is associated with the cognitive decline [18]. The increase in EEG activity during REM sleep may indicate the improvement of brain function.

This conclusion from the FD analysis was supported by conventional EEG spectral analysis. In the present study, we showed that the alpha power increased and the delta/alpha ratio of EEG decreased for REM sleep with CPAP treatment. Previous studies have shown that alpha power belongs to relatively active EEG frequency [6]. And the delta/alpha ratio of EEG has been used to reflect the level of EEG activation [18]. The present study showed the increased alpha power and decreased delta/alpha ratio of EEG, suggesting the increase of EEG activation and improvement of the brain function.

With respect to the zDFD, there were no significant changes after CPAP treatment during both NREM and REM sleep in patients with CSAS, although there was a downward trend. The zDFD is a useful parameter that reflects the variability in EEG complexity. In our previous research, we showed that zDFD decreased remarkably after CPAP therapy in patients with OSAS [9]. The effect of CPAP on the zDFD was different between patients with CSAS and those with OSAS, which may be due to the fact that primary CSAS is a rare disease [15]. Only nine cases of primary CSAS were enrolled in this study, which is a relatively small number of cases. It may also be related to the different impact of CPAP on central and obstructive apnea events, which may require further exploration.

The main limitation of our study was the small sample number of primary CSAS patients due to its low prevalence. In addition, the overall results of the effect of CPAP on CSAS EEG were similar to those of OSA. Whether this result suggests a similar effect of CPAP on OSA and CSAS EEG or a bias due to the small sample size remains to be determined.

## 5. Conclusions

In this study, we have explored, for the first time, the effect of CPAP therapy on sleep EEG characteristics in patients with primary CSAS using FD. With CPAP treatment, the FD significantly decreased in NREM sleep but increased in REM sleep in patients. This suggests that CPAP could reduce the sleep EEG complexity in NREM sleep and increase the sleep EEG activity in REM sleep in patients with CSAS, which may be one of the mechanisms by which CPAP improves brain function in patients with CSAS. Therefore, FD may be used as a new biomarker of electroencephalographic stability and improvement in brain function with CPAP treatment for primary CSAS.

## Figures and Tables

**Table 1:** The demographic and basic data for the nine CSAS participants.

Number of CSAS participants	Age (y)	Gender	BMI (kg/m^2^)	BP before bedtime (mmHg)	BP after sleep (mmHg)	AHI (hr)	AHI on CPAP (hr)
1	40	Male	30	120/90	140/100	37.3	7.2
2	51	Male	35.9	120/85	138/100	84.7	1.3
3	56	Male	26	130/70	140/70	67.5	7.5
4	57	Male	30.8	130/85	140/100	64.8	7.8
5	59	Female	27.8	130/80	150/100	76.5	1.6
6	67	Male	31.1	140/57	160/56	63.9	1.2
7	68	Male	23.4	140/85	120/85	33.9	0.6
8	69	Male	26.4	130/90	130/90	68.5	23.3
9	75	Male	23.8	144/86	160/80	51	3.5

BP, blood pressure; AHI, apnea and hypopnea index; CPAP, continuous positive airway pressure.

**Table 2:** The proportion of sleep in different stages of patients with CSAS before and after continuous positive airway pressure (CPAP) treatment.

	REM%	*N*1%	*N*2%	*N*3%
Before CPAP	8.40 ± 5.32	26.66 ± 16.62	55.13 ± 10.42	9.81 ± 10.62

After CPAP	23.60 ± 9.76^*∗*^	7.38 ± 5.56^*∗*^	49.93 ± 14.40	19.13 ± 15.17

^*∗*^ *p* < 0.05 vs. before CPAP. CSAS, central sleep apnea syndrome; CPAP, continuous positive airway pressure; REM, rapid eye movement.

**Table 3:** The data of FD and zDFD before and after continuous positive airway pressure (CPAP) treatment in patients with central sleep apnea syndrome (CSAS).

CSAS	NREM		REM	
	Before CPAP	After CPAP	Before CPAP	After CPAP
FD	561.67 ± 44.19	438.67 ± 120.83^*∗*^	51.55 ± 31.02	132.81 ± 50.54^*∗*^

zDFD	0.31 ± 0.10	0.25 ± 0.60	0.28 ± 0.19	0.25 ± 0.12

^*∗*^ *p* < 0.05 vs. data before CPAP. FD, fractal dimension; zDFD, zero-crossing rate of detrended FD; CPAP, continuous positive airway pressure; CSAS, central sleep apnea syndrome.

## Data Availability

The data used to support the findings of this study are available from the corresponding author upon request.
